# Efficient Heat Dissipation and Cyclic Electron Flow Confer Daily Air Exposure Tolerance in the Intertidal Seagrass *Halophila beccarii* Asch

**DOI:** 10.3389/fpls.2020.571627

**Published:** 2020-11-30

**Authors:** Yang Fang, Zhijian Jiang, Chunyu Zhao, Linglan Li, Chanaka Isuranga Premarathne Maha Ranvilage, Songlin Liu, Yunchao Wu, Xiaoping Huang

**Affiliations:** ^1^Key Laboratory of Tropical Marine Bio-resources and Ecology, South China Sea Institute of Oceanology, Chinese Academy of Sciences, Guangzhou, China; ^2^University of Chinese Academy of Sciences, Beijing, China; ^3^Southern Marine Science and Engineering Guangdong Laboratory, Guangzhou, China; ^4^Innovation Academy of South China Sea Ecology and Environmental Engineering, Chinese Academy of Sciences, Guangzhou, China; ^5^College of Resources Environment and Planning, Dezhou University, Dezhou, China

**Keywords:** air exposure, intertidal seagrass, photoprotective mechanisms, non-photochemical quenching, cyclic electron flow

## Abstract

Seagrasses inhabiting the intertidal zone experience periodically repeated cycles of air exposure and rehydration. However, little is known about the photoprotective mechanisms in photosystem (PS)II and PSI, as well as changes in carbon utilization upon air exposure. The photoprotective processes upon air exposure in *Halophila beccarii* Asch., an endangered seagrass species, were examined using the Dual-PAM-100 and non-invasive micro-test technology. The results showed that air exposure enhanced non-photochemical quenching (NPQ) in both PSII and PSI, with a maximum increase in NPQ and Y(ND) (which represents the fraction of overall P700 that is oxidized in a given state) of 23 and 57%, respectively, resulting in intensive thermal energy dissipation of excess optical energy. Moreover, cyclic electron transport driven by PSI (CEF) was upregulated, reflected by a 50 and 22% increase in CEF and maximum electron transport rate in PSI to compensate for the abolished linear electron transport with significant decreases in pmf_LEF_ (the proton motive force [pmf]) attributable solely to proton translocation by linear electron flow [LEF]). Additionally, H^+^ fluxes in mesophyll cells decreased steadily with increased air exposure time, exhibiting a maximum decrease of six-fold, indicating air exposure modified carbon utilization by decreasing the proton pump influxes. These findings indicate that efficient heat dissipation and CEF confer daily air exposure tolerance to the intertidal seagrass *H. beccarii* and provide new insights into the photoprotective mechanisms of intertidal seagrasses. This study also helps explain the extensive distribution of *H. beccarii* in intertidal zones.

## Introduction

Seagrass ecosystems have a global distribution, offering indispensable ecosystem services (Hemminga and Duarte, 2000; Barbier et al., 2011), including carbon sequestration (Duarte and Krause-Jensen, 2017), sediment stabilization (Bos et al., 2007), and provision of food and habitats, as well as the reduction of pathogenic microorganisms (Lamb et al., 2017). Seagrasses inhabit both inter- and subtidal coastal zones. Intertidal seagrasses, the focus of this study, are periodically exposed to air during low tide. This frequent tidal exposure could result in desiccation, excessive light, and high-temperature stress in such intertidal seagrasses (Kohlmeier et al., 2017; Manassa et al., 2017; Suykerbuyk et al., 2018). Therefore, the ability of seagrasses to tolerate air exposure and regain competence upon rehydration probably determines their distribution in the intertidal zone.

A diversity of photoprotective mechanisms during air exposure has been described in other intertidal plants. In most cases, non-photochemical quenching (NPQ), photosystem (PS)I-driven cyclic electron flow (CEF), and energy redistribution between PSI and PSII play critical roles in photosynthetic plasticity, allowing the plants to survive environmental stress during air exposure (Gao et al., 2011; Zia et al., 2016; Tan et al., 2017). NPQ is a method for heat dissipation associated with quenching of excitation energy similar to overloading optical energy (Müller et al., 2001; De Carvalho et al., 2011; Yamakawa et al., 2012; Yamakawa and Itoh, 2013). It mainly involves a low thylakoid lumen pH- and zeaxanthin-dependent quenching mechanism (Xu et al., 2015). It has been reported that carotenoids and zeaxanthin that accumulate upon desiccation are crucial to the activation of NPQ, as well as protection of the thylakoid membranes from peroxidation (Havaux et al., 2007; Du et al., 2010; Beckett et al., 2012). Additionally, another protection mechanism mentioned above is involved in the compensation for abolished linear electron flow (LEF) (Gao et al., 2011). CEF promotes electron transfer from the acceptor side of PSI back to plastoquinone, thus maintaining ATP production and supplying the proton gradient (Fan et al., 2016; Yamori and Shikanai, 2016). Therefore, it has been proposed that both NPQ and CEF contribute largely to the protective and adaptive mechanisms of intertidal plants (Gao et al., 2011; Zia et al., 2016; Tan et al., 2017).

In previous studies, considerable attention has been paid to the desiccation-tolerant mechanism in seagrasses (Björk et al., 1999; Shafer et al., 2007; Jiang et al., 2014). However, very few studies have attempted to assess the role of cyclic electron transport driven by PSI in protecting the photosystem (Kohlmeier et al., 2017; Manassa et al., 2017; Suykerbuyk et al., 2018). Additionally, it is not yet known whether there is a tradeoff between PSI and PSII photoprotection for seagrasses. Furthermore, elevated gaseous CO_2_ concentration with less water resistance during air exposure may greatly affect the carbon utilization in seagrass because of the substantial changes from those of the submerged environment, with a lack of instantaneous parameters to confirm it (Silva et al., 2005; Clavier et al., 2011).

The seagrass *Halophila beccarii* Asch, one of the two species in the oldest lineage of seagrasses and listed as a vulnerable species on the IUCN Red List of threatened species (Short et al., 2011), has a relatively large range, with a patchy distribution in the intertidal areas of the tropical Indo-Pacific region (Aye et al., 2014). Recently, the broader distribution of *H. beccarii* was found in the intertidal zone along the coastline of South China (Jiang et al., 2017, 2020). *H. beccarii* in the high intertidal area has even been associated with land plants (Supplementary Figure 1) and endures long-term air exposures. *H beccarii* may show unique photoprotective mechanisms during air exposure. It has been reported that NPQ and cyclic electron transport play critical roles for photoprotection in intertidal plants (Gao et al., 2011; Zia et al., 2016; Tan et al., 2017). However, it is not yet known whether NPQ and cyclic electron transport confer daily air exposure tolerance in the intertidal seagrass *H. beccarii*. Furthermore, it is not known whether HCO_3_^–^ utilization is lowered due to the higher CO_2_ concentration with less transport resistance.

Aiming to close some of these gaps of knowledge about intertidal seagrasses, we conducted an indoor experiment to culture the seagrass *H. beccarii* with different periods of daily air exposure to investigate the photosynthetic, morphological, and biochemical responses to air exposure. Furthermore, we compared instant air exposure with long-term daily air exposure to determine whether long-term adaptation is necessary for photoprotective mechanisms. *In vivo* chlorophyll fluorescence and the P700 redox state of leaves were measured using the Dual-PAM-100. Additionally, measurements of the dual-beam 550- to 515-nm signals simultaneously with the Dual-PAM-100 P515/535 served to identify module proton motive force (pmf) and ATP-synthase activity. Moreover, the net H^+^ flux at mesophyll cells of *H. beccarii* was detected using non-invasive micro-test technology to determine the changes in carbon utilization (Beer et al., 2014). Our hypotheses include the following: (1) photoprotective processes in *H. beccarii* require long-term adaptation to air exposure; (2) enhanced NPQ together with elevated CEF could contribute substantially to photoprotection during air exposure; (3) upon air exposure, leaves would downregulate H^+^ fluxes at mesophyll cells to lower HCO_3_^–^ utilization, based on a tradeoff between elevated gaseous CO_2_ concentration and the reduced demand for carbon sources.

The results obtained in this study will undoubtedly strengthen our understanding of the photosynthetic plasticity and adaptive mechanisms of intertidal seagrasses.

## Materials and Methods

### Plant Materials and Experiment Design

**FIGURE 1:**
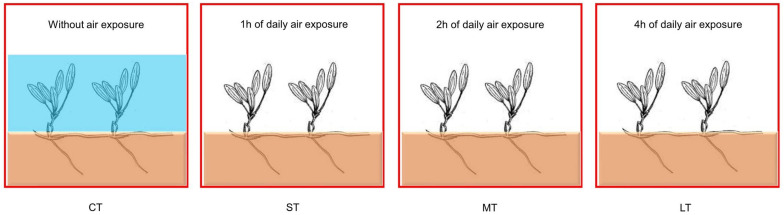
Experimental set-up of the laboratory treatment. CT: Control group without any air exposure; ST: 1 h of daily air exposure; MT: 2 h of daily air exposure; LT: 4 h of daily air exposure.

*Halophila beccarii* was collected with its natural sediment using a PVC corer at the intertidal zone of the monospecific seagrass bed in Yifengxi, along the South China coast (Jiang et al., 2020). Following collection, seagrasses were placed into 16 glass tanks (290 × 160 × 190 mm) with natural seawater in a closed system. For the laboratory acclimation, seagrasses were cultured at 80 μmol photos m^–2^ s^–1^ to simulate natural light intensity for 1 week. After initial laboratory acclimation, *H. beccarii* was cultured with different periods of daily air exposure, including a CT (control group without any air exposure), ST (short time, 1 h of daily air exposure), MT (middle time, 2 h of daily air exposure), and LT (long time, 4 h of daily air exposure) (Figure 1) to imitate the tidal exposure from the high tide area to the low tide area. The average air temperature and humidity were maintained at 20°C and 60%, respectively. In this study, air exposure treatment did not simulate changing temperatures or light intensity. The experimental design emphasized the effect of desiccation, which was in agreement with natural observations. According to the statistical analysis of low tide when seagrasses emerged during 2018–2019 (Supplementary Figure 2), the exact timing at low tide in the daytime primarily occurred between 5:00 and 8:00 a.m. or between 7:00 and 9:00 p.m.; at these times, intertidal exposure to irradiance and the air temperature was expected to be relatively low. After 4 weeks of daily tidal simulation treatment, photosynthetic and other physiological parameters of *H. beccarii* were detected. In particular, we also measured the photosynthetic parameters of seagrass in the CT with instant air exposure of 2 h (Ins-2 h) and 4 h (Ins-4 h), to compare the response mechanism with that of the long-term daily air exposure.

### Chlorophyll Fluorescence and P700^+^ Signal Measurement

*In vivo* chlorophyll fluorescence was measured using the Dual-PAM-100 (Heinz Walz, Effeltrich, Germany). Plants were dark-adapted for 30 min before measurement, and three randomly chosen leaves were detached. The actinic light for measurements of Chl fluorescence was 131 μmol photons m^–2^ s^–1^ (635 nm). The chlorophyll fluorescence parameters were calculated as follows: F_*v*_/F_*m*_ = (F_*m*_–F_*o*_)/F_*m*_, Y(II) = ΦPS II = (F_*m*_′–F_*s*_)/F_*m*_′ = Fq′/Fm′, NPQ = (F_*m*_–F_*m*_′)/F_*m*_′ = F_*m*_/F_*m*_′–1, Y(NPQ) = F/F_*m*_′–F/F_*m*_, Y(NO) = F/F_*m*_ (Lu et al., 2020). F_*o*_ and F_*m*_ are the minimum and maximum fluorescence after dark-adaptation, respectively. F_*o*_′ and F_*m*_’ represent the minimum and maximum fluorescence after light adaptation, respectively. F_*s*_ is the light-adapted, steady-state fluorescence. For PSI: Y(I) = (P_*m*_′–P)/P_*m*_, Y(NA) = (P_*m*_–P_*m*_′)/P_*m*_, Y(ND) = P/P_*m*_ (Baker, 2008; Sun et al., 2020). The CEF value was estimated as the electron transfer rate ETRI-ETRII (Lu et al., 2020).

The redox state of P700 was determined *in vivo* using automated routines provided by the Dual-PAM software (Schreiber, 2008; Lu et al., 2020). The P700 signal was measured during a single turnover flash (200 000 μmol photons m^–2^s^–1^, 10 μs, PQ pools being oxidized) followed by multiple turnover flashes (20 000 μmol photons m^–2^s^–1^, 2 ms, PQ pools were fully reduced) in the presence of a far-red (FR) background light. The change in the P700 signal reflected the dynamic of the P700 oxidation state after FR light exposure and P700 reduction after removal of the FR light. Balancing and calibration of the P700 signal using the automated routine of the Dual-PAM-100 software were performed before each measurement. Kinetic measurements of the dark re-reduction of P700^+^ after turning off the FR light were used for estimation of the half-time (t_1__/__2_) of dark decay of the P700^+^ signal.

### Measurement of Fast Chlorophyll Fluorescence Induction Kinetics Curves

The fast induction kinetics of chlorophyll fluorescence were monitored using the Dual-PAM-100 (Heinz Walz, Effeltrich, Germany). All formulae and glossary of terms used in the JIP-test in the analysis of the O-J-I-P fluorescence transient are presented in Supplementary Table 1. They were used to analyze the changes in the donor side, receptor side, and reaction center of PSII (Straaer, 1995; Stirbet, 2011).

### Measurement of Rapid Light Curves

Rapid light curves (RLCs) of rETR (relative electron transport rate through PSII and PSI) versus irradiance were obtained by exposing leaves to a range of light intensities (13 steps) from 0 to 610 μmol m^–2^ s^–1^. The ETR_max_ (maximum electron transport rate), photosynthetic light-harvesting efficiency (the initial slope of the RLC, α), and l_k_ (half-saturation light intensity) were derived by fitting the RLCs to the equation (Eilers and Peeters, 1988), which was available in the Dual-PAM software.

### Proton Motive Force Measurement

The dual-beam 550- to 515-nm signals were monitored simultaneously using the Dual-PAM-100 P515/535 module. All samples were dark-adapted overnight. Dark interval relaxation kinetics were analyzed to calculate the proton gradient (ΔpH), membrane potential (ΔΨ), and the proton conductivity of the thylakoid membrane (gH^+^) (Sun et al., 2020; Tan et al., 2020). Additionally, pmf_LEF_ (the pmf attributable solely to proton translocation by LEF) was calculated using the following equation: pmf_LEF_ = LEF/gH^+^, in which was used in the estimation of the light-driven proton flux through the ATP synthase based on the extent of LEF and the kinetic properties of the ATP-synthase turnover (Avenson et al., 2005).

### Measurement of H^+^ Fluxes at Mesophyll Cells

*In vivo* net H^+^ fluxes at mesophyll cells were measured with a non-invasive micro-test technology (NMT, Younger, Amherst, MA, United States; Xuyue Science and Technology Co., Ltd., Beijing, China). Washed leaves were cut in half and incubated in the standard medium buffer (100 mM NaCl) for 5 min at room temperature. Microelectrodes were positioned close to the mesophyll cells. Finally, we entered the data Origin (1) millivolts (mV) and AvgOrigin-X microvolts (μV) as measured by the ASET 2.0 software (The imFlux^®^ Software). H^+^ flux was then calculated directly by using the JCal v3.2.1 software. Each sample was monitored for the kinetics of net H^+^ fluxes for 10 min. Three biological replicates were performed per group.

### Chemical and Morphological Analysis

The content of chlorophyll and carotenoids in leaves was determined by a simplified spectrophotometry method (Shu et al., 2010). A leaf with known weight was extracted overnight in 5 mL of 80% acetone solution, and the volume of the extracted solution was determined in 5 mL. The optical density was read at 663, 645, and 470 nm, respectively, by spectrophotometry, and the content of photosynthetic pigments was calculated by the following formulae:

Chl a (μg mL^–1^) = 12.7 OD_663_–2.69 OD_645_

Chl b (μg mL^–1^) = 22.9 OD_645_–4.86 OD_663_

Carotenoid (μg mL^–1^) = (OD_470_–3.27 Chl a–104 Chl b)/229

Leaf length, leaf width, and root length were measured for five randomly selected leaves from each group. Seagrasses were carefully retrieved, separated into aboveground and belowground tissues, and subsequently dried at 60°C for 72h until a constant weight was achieved. Seagrasses were then homogeneously powdered. The total carbon and nitrogen of seagrasses were analyzed using an Elementary Analyzer (Flash EA 3000, Thermo Scientific, Milan, Italy).

### Statistical Analysis

The measurements were conducted on randomly selected samples, and the values presented are the mean ± SD of a minimum of three replicates. The data were analyzed using SPSS 23 Software (IBM SPSS Statistics, Armonk, NY, United States) using ANOVA, and a statistically significant difference was set at a probability level of 0.05. LSD (least significant difference)-*post hoc* tests were performed to evaluate *post hoc* pairwise comparisons. The figures were drawn with Origin 9 Software (Origin Lab, Northampton, MA, United States).

## Results

### Responses of ETR and CEF

**FIGURE 2:**
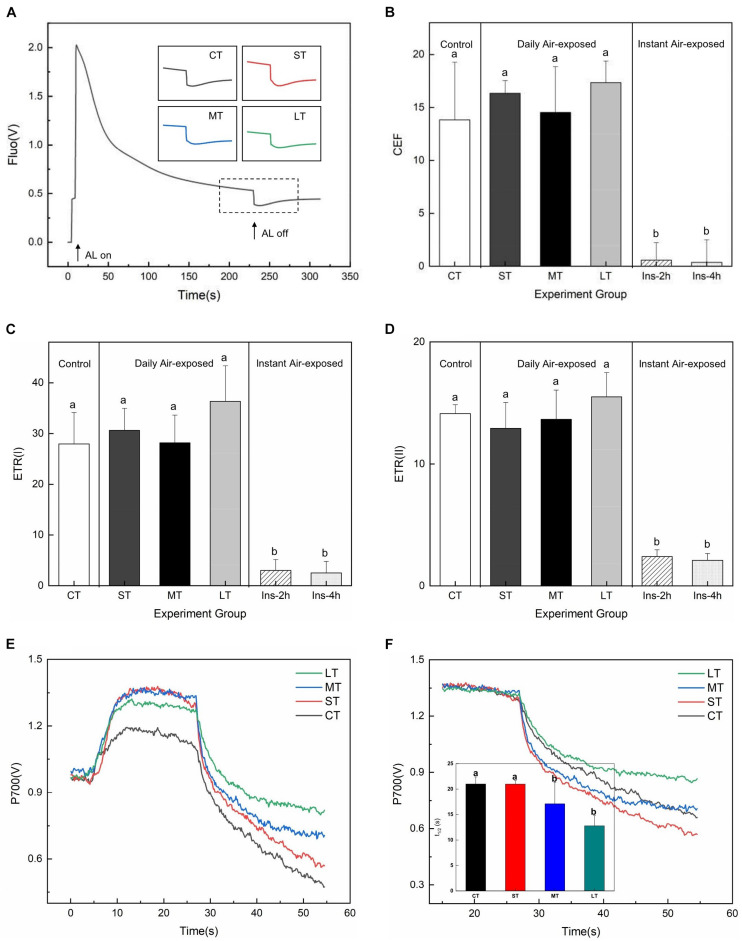
Effects of air exposure treatments on ETR and cyclic electron flow. **(A)** Post-illumination increases of chlorophyll fluorescence in the treatments. **(B)** Changes in CEF in the treatments. **(C)** Changes in ETR(II) in the treatments. **(D)** Changes in ETR(I) in the treatments. **(E)** The P700 signal in the treatments. **(F)** Enlarged display of P700 signal after FR light was removed and the half (t_1__/__2_) of dark decay kinetics to the steady state estimated from the P700 signal. All data are expressed as means ± SD based on experiments in triplicate. Different letters over columns in panels **(B–D,F)** indicate significant difference (*P* < 0.05) among means. CT: Control group without any air exposure; ST: 1 h of daily air exposure; MT: 2 h of daily air exposure; LT: 4 h of daily air exposure; Ins-2 h: instant air exposure of 2 h; Ins-4 h: instant air exposure of 4 h.

Our results demonstrated that the increase in transient post-illumination did not affect chlorophyll fluorescence when subjected to air exposure treatment (Figure 2A). Daily air exposure did not affect CEF, although instant air exposure decreased significantly (*Fa,b* = 16.03, *n* = 18, *P* < 0.01) (Figure 2B). Similar trends were observed in both ETR(I) (*Fa,b* = 27.07, *n* = 18, *P* < 0.01) (Figure 2C) and ETR(II) (*Fa,b* = 43.941, *n* = 18, *P* < 0.01) (Figure 2D), strongly indicating attenuated electron transfer in instant air-exposed leaves. In contrast, daily air-exposed leaves showed considerably higher CEF and ETR than did the instant air-exposed leaves.

Daily air exposure also enhanced the maximum P700^+^ signals (P700 fully oxidized) of leaves (Figure 2E). The half-time (t_1__/__2_) of dark decay kinetics to the steady-state (re-reduction of P700^+^) after turning off the FR light declined with the increase in daily air-exposed hours (*Fa,b* = 7.615, *n* = 18, *P* < 0.05) (Figure 2F), suggesting exacerbated CEF driven by PSI.

**FIGURE 3:**
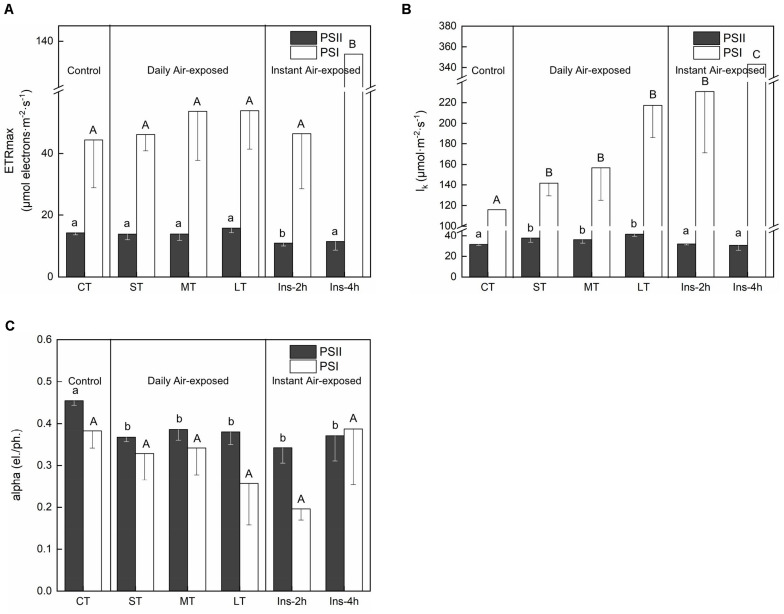
Rapid light curves fitting parameters under different air exposure treatments. **(A)** ETR_max_ estimated in PSII and PSI. **(B)** l_k_ (half-saturation light intensity) estimated in PSII and PSI. **(C)** Alpha (initial slope of fitting curve) estimated in PSII and PSI. All data are expressed as means ± SD based on experiments in triplicate. Different letters over the columns indicate significant difference (*P* < 0.05) among means. Upper- and lower-case letters are used to distinguish different parameters within each figure part. CT: Control group without any air exposure; ST: 1 h of daily air exposure; MT: 2 h of daily air exposure; LT: 4 h of daily air exposure; Ins-2 h: instant air exposure of 2 h; Ins-4 h: instant air exposure of 4 h.

ETR_max_ from the curve fitting for RLCs showed distinctly changing trends in PSII and PSI. Daily air exposure did not affect ETR_max_ of PSII, whereas instant air exposure considerably decreased it (*Fa,b* = 3.347, *n* = 18, *P* < 0.05) (Figure 3A). Similarly, only instant air exposure significantly enhanced ETR_max_ in PSI (*Fa,b* = 4.918, *n* = 18, *P* < 0.05) (Figure 3A).

Concomitantly, daily air exposure markedly promoted l_k_ (half-saturation light intensity) in PSII, whereas instant air exposure showed no effect (*Fa,b* = 5.824, *n* = 18, *P* < 0.01). However, daily and instant air exposure both enhanced l_k_ in PSI (*Fa,b* = 14.403, *n* = 18, *P* < 0.01), with greater enhancement by instant air exposure (Figure 3B).

Similarly, daily and instant air exposure both significantly decreased the alpha (initial slope of the fitting curve) of PSII (*Fa,b* = 3.876, *n* = 18, *P* < 0.05) (Figure 3C), although a non-significant trend was observed in PSI with a given decrease in daily air-exposed leaves (*Fa,b* = 2.674, *n* = 18, *P* > 0.05).

### Response of PSII Activity

No significant difference was observed in maximal quantum yield of PSII (F_*v*_/F_*m*_) among treatments (Table 1) (*Fa,b* = 0.572, *n* = 18, *P* > 0.05). Only instant air exposure markedly stimulated Y(II) (*Fa,b* = 90.228, *n* = 18, *P* < 0.01), Y(NO) (*Fa,b* = 9.059, *n* = 18, *P* < 0.01) and relative Q_*A*_ reduction (*Fa,b* = 9.248, *n* = 18, *P* < 0.01), but reduced Y(NPQ) (*Fa,b* = 180.521, *n* = 18, *P* < 0.01) and NPQ (*Fa,b* = 51.722, *n* = 18, *P* < 0.01).

**TABLE 1:** Variation in PSII activity during daily and instant air exposure treatment (values are means of three replicates ± SD).

	F_*v*_/F_*m*_	Y(II)	Y(NPQ)	Y(NO)	NPQ	Relative Q_*A*_ Reduction
CT	0.79 ± 0.01^ns^	0.26 ± 0.01^a^	0.45 ± 0.01^a^	0.29 ± 0.01^a^	1.55 ± 0.02^a^	0.29 ± 0.01^a^
ST	0.78 ± 0.02^*ns*^	0.24 ± 0.04^a^	0.50 ± 0.05^a^	0.27 ± 0.03^a^	1.88 ± 0.37^a^	0.27 ± 0.03^a^
MT	0.79 ± 0.01^ns^	0.25 ± 0.04^a^	0.46 ± 0.01^a^	0.29 ± 0.03^a^	1.63 ± 0.16^a^	0.29 ± 0.03^a^
LT	0.78 ± 0.00^*ns*^	0.27 ± 0.02^a^	0.48 ± 0.04^a^	0.25 ± 0.01^a^	1.90 ± 0.24^a^	0.27 ± 0.02^a^
Ins-2 h	0.78 ± 0.01^NS^	0.58 ± 0.01^b^	0.08 ± 0.01^b^	0.33 ± 0.01^b^	0.25 ± 0.02^b^	0.34 ± 0.01^b^
Ins-4 h	0.79 ± 0.01^*NS*^	0.58 ± 0.04^b^	0.07 ± 0.01^b^	0.35 ± 0.03^b^	0.20 ± 0.03^b^	0.35 ± 0.03^b^

*CT: control group without any air exposure; ST: 1 h of daily air exposure; MT: 2 h of daily air exposure; LT: 4 h of daily air exposure; Ins-2 h: instant air exposure of 2 h; Ins-4 h: instant air exposure of 4 h.*

Although non-significant, Y(NPQ) and NPQ had higher values in the daily air-exposed leaves than in the control and instant air-exposed leaves, suggesting a higher energy allocation in NPQ. Additionally, daily air-exposed leaves showed lower levels of Y(NO) and relative Q_*A*_ reduction, indicating a greater extent of PSII photoinhibition in instant air-exposed leaves.

**FIGURE 4:**
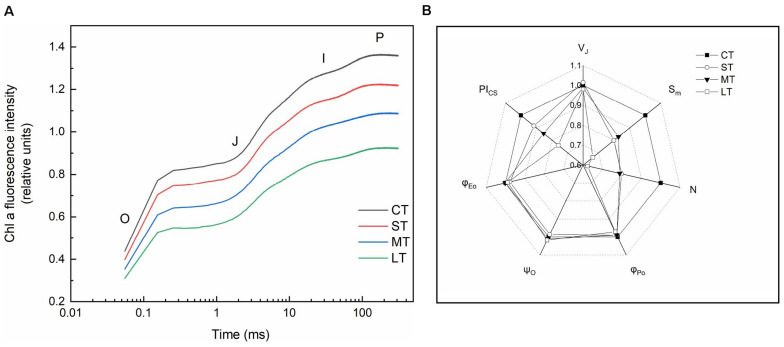
Rapid chlorophyll fluorescence induction kinetics. **(A)** OJIP transient (values are means of three replicates). **(B)** The spider plots of the chlorophyll fluorescence parameters in different treatments. V_J_: Relative variable fluorescence intensity at J point. S_m_: The complementary area between the O-J-I-P induction curve, F = FM and the *Y*-axis. N: The number of times that Q_A_ was restored during the period from the start of illumination to FM. φ_Po_: Maximal photochemical efficiency. ψ_O_: The ratio of the excitons captured by the reaction center to the other electron acceptors used to promote electron transfer to the electron transfer chain, which exceeds Q_A_, to the excitons used to promote Q_*A*_ reduction. φ_Eo_: Quantum yield for electron transfer. PI_CS_: Performance index based on unit area. All data are expressed as means ± SD based on experiments in triplicate. CT: Control group without any air exposure; ST: 1 h of daily air exposure; MT: 2 h of daily air exposure; LT: 4 h of daily air exposure.

Rapid chlorophyll fluorescence induction kinetics curve (OJIP curve) was only detected in daily air-exposed leaves (Figure 4). The changes in the donor side of PSII, the maximum fluorescence intensity (P point), decreased as the air-exposed time increased (Figure 4A). Changes in the receptor side of PSII, the maximal photochemical efficiency (φ_Po_), decreased slightly in air-exposed leaves. The PQ pool decreased, which was reflected by lower S_m_, and further led to a slight decline in φ_Eo_. Restore times of Q_A_ (N) decreased, which was consistent with the decreased ability to transfer electrons. No significant change was observed in the opening degree of the active reaction center (ψ_O_) (Figure 4B). The changes in the reaction center of PSII, the performance index (PIcs), indicated the effect of air exposure on PSII, which was significantly decreased with increased exposure time. There were no significant changes in V_J_, in accordance with the unchanged proportion of inactive reaction centers (Figures 4B, 2B).

### Response of PSI Activity

**FIGURE 5:**
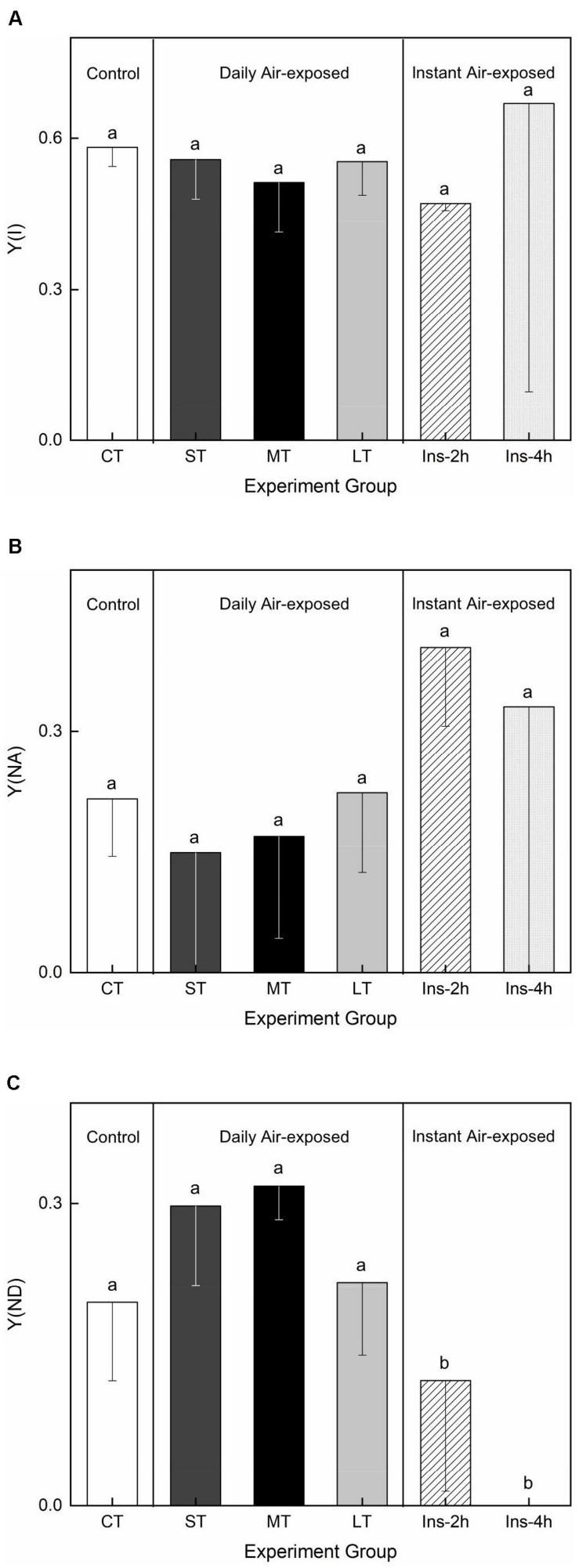
Effects of air exposure treatment on PSI activity. **(A)** Changes in Y(I) in the treatments. **(B)** Changes in Y(NA) in the treatments. **(C)** Changes in Y(ND) in the treatments. All data are expressed as means ± SD based on experiments in triplicate. Different letters over the columns indicate significant difference (*P* < 0.05) among means. CT: Control group without any air exposure; ST: 1 h of daily air exposure; MT: 2 h of daily air exposure; LT: 4 h of daily air exposure; Ins-2 h: instant air exposure of 2 h; Ins-4 h: instant air exposure of 4 h.

Air exposure did not affect Y(I) (*Fa,b* = 0.233, *n* = 18, *P* > 0.05) or Y(NA) (*Fa,b* = 0.449, *n* = 18, *P* > 0.05) (Figures 5A,B). Daily air-exposed leaves showed lower levels of both Y(I) and Y(NA), in contrast to instant air-exposed leaves. Additionally, daily air exposure did not affect Y(ND) (which represents the fraction of overall P700 that is oxidized in a given state), whereas instant air exposure significantly decreased it (*Fa,b* = 8.220, *n* = 18, *P* < 0.01), with Y(ND) in Ins-4 h reaching zero and suggesting serious photoinhibition. In comparison, daily air-exposed leaves showed much higher levels of Y(ND), indicating an increased quantum yield of PSI non-photochemical energy dissipation (Figure 5C).

### Response of Electrochromic Shift (ECS) and H^+^ Fluxes

**FIGURE 6:**
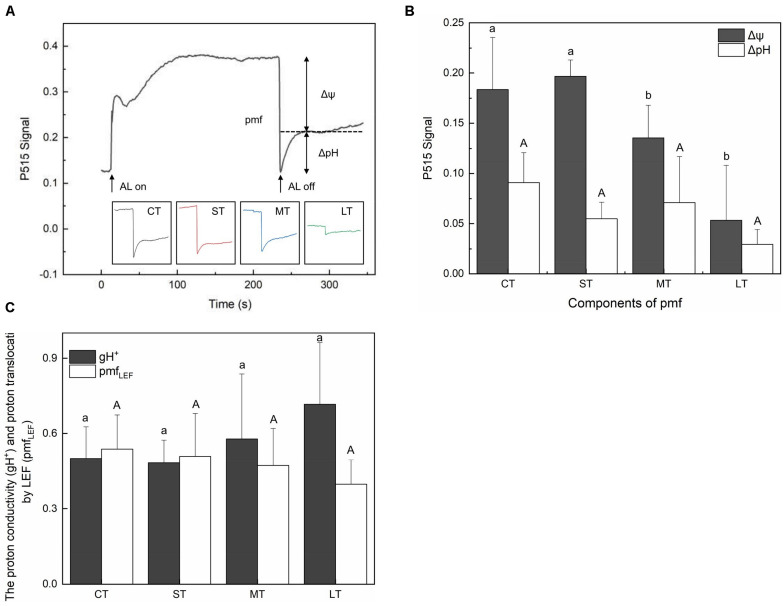
Effects of air exposure treatment on Electrochromic shift (ECS). **(A)** Changes in the P515 signal of slow dark–light–dark induction transients. **(B)** Two components of the proton motive force (membrane potential and proton gradient) derived from the slow dark–light–dark induction transients of the 550 to 515 nm signals. **(C)** Proton conductivity of the thylakoid membrane and the pmf attributable solely to proton translocation by LEF estimated from P515 signal. All data are expressed as means ± SD based on experiments in triplicate. Different letters over columns in panels **(B,C)** indicate significant difference (*P* < 0.05) among means. Upper- and lower-case letters are used to distinguish different parameters in panels **(B,C)**. CT: control group without any air exposure; ST: 1 h of daily air exposure; MT: 2 h of daily air exposure; LT: 4 h of daily air exposure.

The membrane potential (Δψ) and proton gradient (ΔpH) of the pmf in daily air-exposed leaves were estimated by analyzing the light-off responses of the P515 signal (Figure 6A). Significant changes in Δψ were detected (*Fa,b* = 7.195, *n* = 12, *P* < 0.01). A transient increase in Δψ was observed in ST, followed by a decline in Δψ in MT and LT (Figure 5B). Furthermore, though not significant (*Fa,b* = 2.308, *n* = 12, *P* > 0.05), a fluctuating decline in ΔpH is shown in Figure 6B. The pmf was lowest in LT, probably because it had the longest air exposure. Additionally, air exposure did not significantly affect gH^+^ (*Fa,b* = 0.903, *n* = 12, *P* > 0.05) or pmf_LEF_ (*Fa,b* = 0.560, *n* = 12, *P* > 0.05), but a trend was observed. Though not significant, air-exposed leaves showed higher levels of gH^+^ (proton conductivity of the thylakoid membrane). Additionally, the pmf attributable solely to proton translocation by LEF decreased slightly in air-exposed leaves as the exposure time increased (Figure 6C), in accordance with exacerbated CEF (Figure 2B).

**FIGURE 7:**
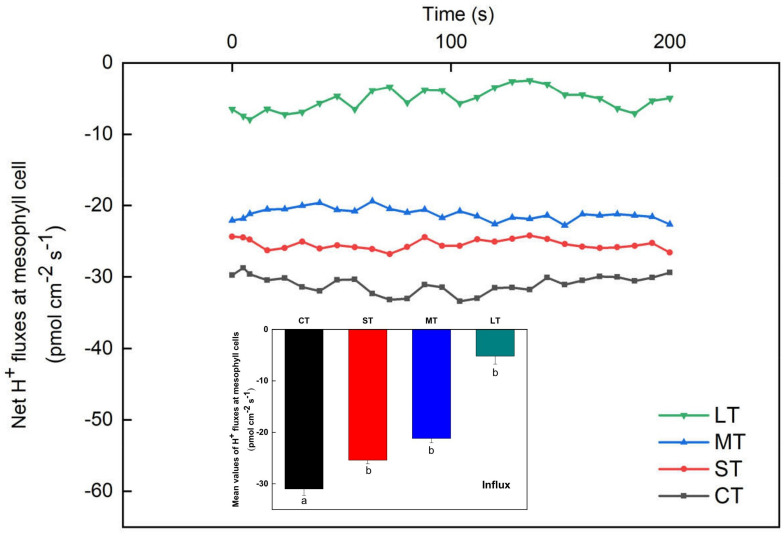
Net H^+^ fluxes at mesophyll cells of *H. beccarii* in different air exposure treatments. All data are expressed as means ± SD based on experiments in triplicate. Different lowercase letters under the four columns indicate significant difference (*P* < 0.05) among means. CT: Control group without any air exposure; ST: 1 h of daily air exposure; MT: 2 h of daily air exposure; LT: 4 h of daily air exposure.

The net H^+^ fluxes at mesophyll cells of *H. beccarii* in different air exposure treatments are depicted in Figure 7. The net H^+^ fluxes under CT, ST, MT, and LT were 31.00 ± 1.24, 25.40 ± 0.70, 21.19 ± 0.85, and 5.18 ± 1.56 pmol cm^–2^ s^–1^, respectively. Air exposure significantly decreased the net H^+^ fluxes (*Fa,b* = 3.936, *n* = 12, *P* < 0.05).

### Changes in Chemical and Morphological Properties

**FIGURE 8:**
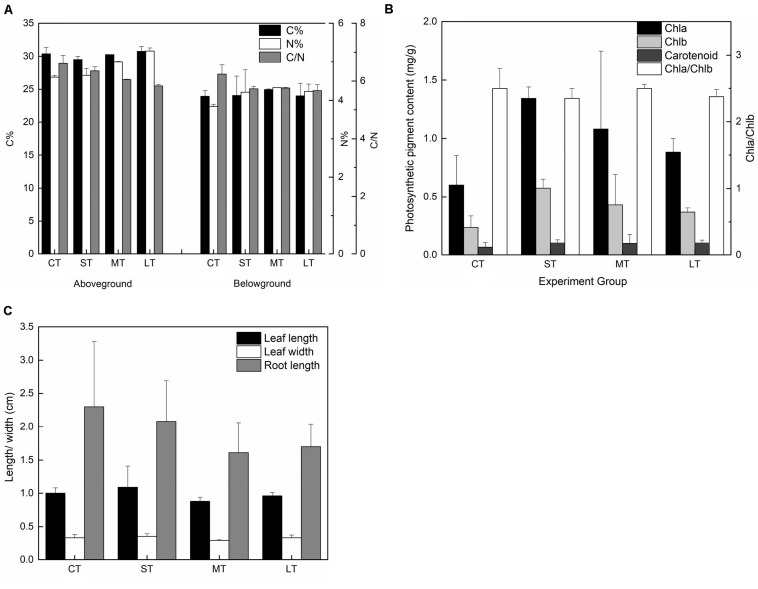
Effects of air exposure on the chemical and morphological changes. **(A)** The changes in carbon and nitrogen content in air exposure treatments (values are means of two replicates). **(B)** The changes in photosynthetic pigments including of Chl a, Chl b, and carotenoids in air exposure treatments (values are means of three replicates). **(C)** The changes in morphology including of leaf length, leaf width, and root length in air exposure treatments. All data are expressed as means ± SD based on experiments in triplicate. CT: Control group without any air exposure; ST: 1 h of daily air exposure; MT: 2 h of daily air exposure; LT: 4 h of daily air exposure.

There were no significant differences in carbon content in both the aboveground (*Fa,b* = 1.247, *n* = 8, *P* > 0.05) and belowground (*Fa,b* = 0.138, *n* = 8, *P* > 0.05) tissues among air exposure treatments (Figure 8A). However, air-exposed leaves showed higher levels of nitrogen content in aboveground and belowground tissues (aboveground: *Fa,b* = 19.258, *n* = 8, *P* < 0.05; belowground: *Fa,b* = 0.997, *n* = 8, *P* > 0.05), resulting in a lower C/N ratio (aboveground: *Fa,b* = 9.008, *n* = 8, *P* < 0.05; belowground: *Fa,b* = 3.728, *n* = 8, *P* > 0.05) (Figure 8A). Furthermore, although non-significant, photosynthetic pigments, including Chl a (*Fa,b* = 2.221, *n* = 12, *P* > 0.05), Chl b (*Fa,b* = 2.740, *n* = 12, *P* > 0.05) and carotenoids (*Fa,b* = 0.430, *n* = 12, *P* > 0.05), increased in all air exposure treatments, whereas the Chl a/Chl b ratio remained stable (*Fa,b* = 0.577, *n* = 12, *P* > 0.05) (Figure 8B).

No statistically significant difference was observed in leaf length (*Fa,b* = 3.002, *n* = 20, *P* > 0.05), leaf width (*Fa,b* = 2.532, *n* = 20, *P* > 0.05), or root length (*Fa,b* = 1.234, *n* = 20, *P* > 0.05). The changing trend in leaf length and width was similar, with an increase in ST and a decrease in MT and LT (Figure 8C). Root length in the air exposure groups was lower compared to that of CT (Figure 8C).

## Discussion

### Efficient Heat Dissipation and Cyclic Electron Flow (CEF) Confer Daily Air Exposure Tolerance

The seagrass *H. beccarii* inhabits the intertidal zones and periodically experiences repeated cycles of air exposure and rehydration. To our knowledge, very few studies have attempted to assess the difference in photosynthetic responses of seagrasses exposed to instant and long-term daily air exposure. In this study, protective processes were found only in daily air-exposed leaves. In contrast, the NPQ of PSII was absent in instant air-exposed leaves, reflected by higher levels of Y(NO) and relative Q_*A*_ reduction. This indicated that instant air exposure caused photodamage and a higher turn-off proportion of the PSII reaction center. Previous studies have indicated that the extent of PSII photoinhibition was dependent on the reduction in Q_*A*_ (Kramer et al., 2004), which could reduce the D1 protein content by recombination with P680^+^ (Krieger-Liszkay et al., 2008; Vass, 2011). It has also been reported that carotenoids, which accumulated upon desiccation, were crucial to activating NPQ in plants (Demmig-Adams, 2003; Du et al., 2010; Beckett et al., 2012). Thus, the deactivated NPQ leading to photoinhibition in instant air-exposed leaves might be caused by low levels of carotenoids. Moreover, CEF, essential for efficient photoprotection (Pinnola and Bassi, 2018; Lu et al., 2020), was also absent in instant air-exposed leaves, which was also reflected in significantly lower CEF and ETR in both PSII and PSI. It should be noted that the distinct difference in photosynthetic physiology between instant and daily air exposure was that daily air exposure allowed the repeated rehydration period to relax. Similar research on the effect of fluctuating light on rice and *Arabidopsis thaliana* suggested that PSI is more sensitive to fluctuating light than to constant high light, and CEF is required to alleviate photodamage of both photosystems by fluctuating light (Kono et al., 2014; Yamori and Shikanai, 2016; Yamori et al., 2016). Therefore, based on our results and published studies, we deduced that only daily air exposure enhanced CEF because of the sensibility of PSI to repeated cycles of air exposure and dehydration.

**FIGURE 9:**
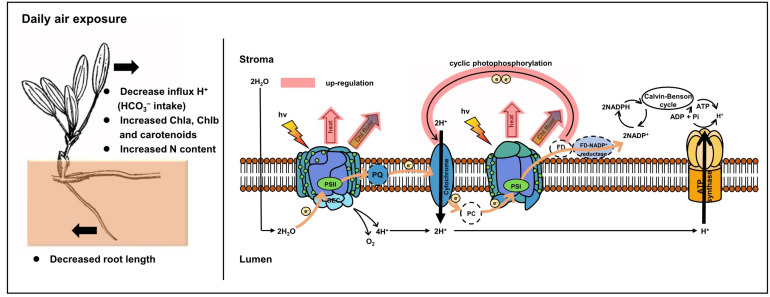
Photoprotective mechanisms in intertidal *H. beccarii* upon daily air exposure. NPQ and CEF indicated in red were upregulated in tolerance with air exposure stress.

In comparison, a protective and adaptive mechanism for mediating daily air exposure stress was observed. It consisted of two main processes (Figure 9). First, NPQ in both PSII and PSI was observed to be exacerbated with higher levels of Y(NPQ) and Y(ND), which resulted in intensive thermal energy dissipation of excess optical energy. Consequently, the activity of PSII and PSI was maintained, as reflected by the unchanged F_*v*_/F_*m*_ and Y(I). The unchanged F_*v*_/F_*m*_ and Y(I) suggested that photosynthetic efficiency was stable at 0 to 4 h of air exposure, which could be explained by the photosynthetic plasticity, as well as flexible leaves that easily make contact on the moist sediment surface. Similar trends were observed in terrestrial plants and aquatic plants under moderate levels of drought or desiccation stress (Bian and Jiang, 2009; Kang et al., 2013; Li et al., 2019). Similar to the previous study, elevated NPQ allowed energy to bypass reaction centers at the beginning of the absorbed excitation energy partition, and it was rapidly converted to thermal energy (Sharkey and Zhang, 2010; Yamakawa et al., 2012; Hernández-Fuentes et al., 2019). Despite the protected photosynthetic active centers, photochemical efficiency was affected under air exposure. Notably, P700^+^ signal and S_*m*_ were observed to decrease, suggesting lower levels of the PQ pool as a consequence of water deficit. Additionally, LEF was inhibited because of limited electron production from water photolysis (Gao et al., 2011), further resulting in lower pmf_LEF_. Second, to compensate for the linear electron transport, the CEF transport driven by PSI was upregulated. The results were higher levels of CEF and ETR_max_ in PSI, as well as lower t_1__/__2_. This was interpreted as elevated CEF, in accordance with decreased pmf_LEF_. A similar study on the seagrasses *Enhalus acoroides* and *Thalassia hemprichii* indicated that enhanced ETR_max_ upon desiccation was consistent with relative water content being higher than the critical threshold, which caused no irreversible damage (Jiang et al., 2014). It is also noteworthy that this experimental design emphasized the effect of desiccation with suitable temperature and light intensity, in accordance with natural observations. Thus, the degree of stress might not yet induce significantly upregulated CEF but induced certain increased trends, which further confirmed a high extent of photosynthetic plasticity. In the following processes, CEF could promote electron transfer from the acceptor side of PSI back to plastoquinone, thus maintaining ATP production and supplying the proton gradient (Fan et al., 2016; Yamori and Shikanai, 2016), consistent with a slightly increased ΔpH of the ST group in this study. The formation of ΔpH was further suppressed with increasing air exposure hours because of the increased thylakoid H^+^ efflux activity from the luminal to the stromal side, reflected by elevated gH^+^. Based on these results, we tentatively propose that cyclic electron transport contributed substantially to air exposure tolerance for intertidal *H. beccarii* (Figure 9), which helped explain the broad distribution of *H. beccarii* in intertidal zones.

### Air Exposure Decreased the Proton Pump Influxes

Seagrass can use CO_2_ and HCO_3_^–^ as inorganic carbon sources, depending on the species (Beer et al., 2014). The pathways of inorganic carbon source utilization in seagrass leaves are quite different from those of other plants because of the absence of stoma on the leaf epidermis. For example, the seagrass *Halophila ovalis*, the same genus as *H. beccarii* in this study, was previously studied using HCO_3_^–^ as a carbon source (Uku et al., 2005). More specifically, protons are extruded by an active H^+^ pump somewhere along the leaf, then transported back into the cells, accompanied by HCO_3_^–^ (Beer et al., 2014). Under high light or drought, plants usually close stoma to reduce water loss by transpiration and decrease the internal leaf CO_2_ concentration (Dubey, 1996; Beer et al., 2014; Zhang et al., 2017). However, for seagrass without the stomatal regulation mechanism, air exposure may increase the photosynthetic rate in seagrass because of the higher ambient CO_2_ concentration (Jiang et al., 2014). However, as exposure time increased, the carbon intake decreased along the gradually rising water loss. In this study, air-exposed leaves made a tradeoff between elevated gaseous CO_2_ concentrations and the reduced demand for carbon sources by downregulating the HCO_3_^–^ intake. More specifically, air-exposed leaves exhibited lower levels of H^+^ fluxes as the exposure time increased. It should be noted that this study has examined only changes in H^+^ fluxes to confirm HCO_3_^–^ utilization. Detection of carbonic anhydrase activity and changes in HCO_3_^–^ concentration in water could further characterize carbon utilization under air exposure (Beer et al., 2014). Notwithstanding its limitation, this study does suggest that proton pump and carbon intake were downregulated.

### Air Exposure Enhanced Photosynthetic Pigments but Reduced Root Length

The present study indicated that photosynthetic pigments, including Chl a, Chl b, and carotenoids, increased in all air exposure treatments (Figure 9). Previous studies proposed that photosynthetic pigments, such as carotenoids and zeaxanthin, were necessary for inducing NPQ, quenching of singlet oxygen, and protecting thylakoid membranes from peroxidation (Havaux et al., 2007; Du et al., 2010; Beckett et al., 2012), in accordance with strengthened NPQ in both PSII and PSI, as mentioned above. Additionally, it was reported that the content of carotenoids in *Boea hygrometrica* was partially increased upon dehydration (Tan et al., 2017), which was similar to the findings in this study. Therefore, based on our results and published studies, we suggest that accumulated photosynthetic pigments in air-exposed leaves contribute to the photoprotective mechanism (Figure 9). It should be noted that this study has only detected the total carotenoid content without quantifying zeaxanthin or violaxanthin, which merits further study. In addition to the photosynthetic pigment discussed in this study, it would also be worthwhile to study the role of secondary metabolites in plants, including flavonoids and phenols that could play a role in the protection of photoinhibition against high irradiance and increased temperature during air exposure (Close and Beadle, 2003; Abdala-Díaz et al., 2006). This requires further investigation.

Although morphological changes in this study were not statistically significant, there were some interesting findings. The changing trend in leaf length and width was similar, with a rise in 1 h long-term exposure and a drop in 2 h and 4 h long-term exposure. The leaf change trend of being smaller was similar in other seagrass species (*Zostera marina*; *Zostera noltii*; *Zostera japonica*) inhabiting high tide zones (Harrison, 1982; Short and Burdick, 1996; Invers et al., 2004; Heck and Valentine, 2006). It seems possible that smaller leaves are more flexible to maintain contact with the moist sediment surface to reduce water loss. Additionally, the energy cost for both shedding damaged leaves upon desiccation and growing new leaves under suitable conditions would be much lower with smaller leaves, which are utilized by *Ruppia cirrhosa* and *Zostera capensis* to rapidly recover from desiccation stress (Pérez-Lloréns and Niell, 1993). Similar changes were also found in the response mechanism of leaf abscission in land and floating plants under water deficit (McMichael et al., 1973; Adams and Bate, 1994; Beer and Bjork, 2000; Saltmarsh et al., 2006). Furthermore, stress-mediated reduction in cell turgor of the meristem could lead to suppressed cell division and elongation (Nelissen et al., 2018; Todaka et al., 2019). Regarding the observed changes in root length in this study, it was surprising that root length in air exposure groups decreased because longer roots might benefit from drawing water from the deeper sediment. It has been reported that increased rooting depth is often observed as a primary response to drought in terrestrial plants (Kashiwagi et al., 2005; Comas et al., 2013). While considering the different aquatic habitat of seagrass, the water content of surface sediment is higher; thus, root elongation for drawing water may be unnecessary. Therefore, decreased root length was probably caused by attenuated carbon assimilation during the air exposure period. However, higher efficiency of carbon assimilation for submerged seagrass, in accordance with the higher pmf, might lead to longer roots by providing a greater carbon source. Further long-term research on root growth and turnover is needed to investigate seagrass plasticity under air exposure. Moreover, no differences in total carbon content and slightly higher levels of total nitrogen in exposed leaves were observed, resulting in a lower C/N ratio (Figure 9). This is seen as a consequence of the gradual dilution of stored nutrient resources in the control group because of a high growth rate (Peralta et al., 2002; Jiang et al., 2010). Lower carbon assimilation upon air exposure with smaller seagrass leaves might also cause non-change of total carbon content. Furthermore, free amino acid accumulation, such as proline, is needed to adjust osmotic pressure to enhance tolerance to desiccation (Pandey et al., 2010; Pizarro et al., 2019), which might explain the increased nitrogen accumulation.

## Conclusion

In summary, our results indicated that two main photoprotective mechanisms found in the intertidal seagrass *H. beccarii* required long-term adaptation to daily air exposure. Efficient NPQ in both PSII and PSI resulted in intensive thermal energy dissipation of excess optical energy upon air exposure. CEF driven by PSI was upregulated to compensate for the abolished linear electron transport, although the role of water–water cycles in tolerating daily air exposure warrants further research (Huang et al., 2019). More detailed quantification of zeaxanthin and violaxanthin could further determine the role of photosynthetic pigments in photoprotective processes. Additionally, H^+^ fluxes at mesophyll cells were downregulated for lower inorganic carbon uptake. Furthermore, modern approaches based on transcriptomics, proteomics, and metabolomics will hopefully be involved in investigations on molecular mechanisms of desiccation-tolerant seagrass in intertidal zones in future research.

## Data Availability Statement

The original contributions presented in the study are included in the article/Supplementary Material, further inquiries can be directed to the corresponding author/s.

## Author Contributions

XH and ZJ designed the study. YF, CZ, LL, CR, SL, and YW performed the experiments. YF, ZJ, and XH analyzed the data and wrote the manuscript. All authors contributed to the article and approved the submitted version.

## Conflict of Interest

The authors declare that the research was conducted in the absence of any commercial or financial relationships that could be construed as a potential conflict of interest.

## Supplementary Material

Supplementary Figure 1 *H. beccarii* associated with the land plant in the high intertidal area.

Supplementary Figure 3 Statistical analysis of the exact timing and tidal height of low tide when seagrasses can emerge in Yifengxi, along the South China coast, during 2018–2019.

Supplementary Table 1 Formulae and glossary of terms used in the JIP test in the analysis of the O-J-I-P fluorescence transient. CT: control group without any air exposure; ST: 1 h of daily air exposure; MT: 2 h of daily air exposure; LT: 4 h of daily air exposure.
