# Supplementary material for: Efficient Heat Dissipation and Cyclic Electron Flow Confer Daily Air Exposure Tolerance in the Intertidal Seagrass Halophila beccarii Asch
Source: Front Plant Sci. 2020 Nov 30;11:571627. doi: 10.3389/fpls.2020.571627 (PMC7733926; doi:10.3389/fpls.2020.571627)
Supplement: Supplementary Figure 1 — H. beccarii associated with the land plant in the high intertidal area. [file Data_Sheet_1.PDF]

Supplementary Material

SUPPLEMENTARY TABLES AND FIGURES

Table S1. Formulae and glossary of terms used in the JIP-test in the analysis of the O-J-I-P fluorescence transient

Formulae and glossary	Definition
$F_O \equiv F_{0ms}$	Fluorescence intensity at O (0 ms) of the O-J-I-P induction curve
$F_J \equiv F_{2ms}$	Fluorescence intensity at J (2 ms) of the O-J-I-P induction curve
$F_I \equiv F_{30ms}$	Fluorescence intensity at I (30 ms) of the O-J-I-P induction curve
F_P	The maximum fluorescence intensity of the O-J-I-P induction curve
$V_J \equiv (F_J - F_O)/(F_M - F_O)$	Relative variable fluorescence intensity at J
$M_O \equiv 4(F_{300\mu s} - F_O)/(F_M - F_O)$	initial slope of the O-J-I-P induction curve
$S_m \equiv (Area)/(F_M - F_O)$	The complementary area between the O-J-I-P induction curve, F = FM and the Y-axis
$S_S = V_J/M_O$	Fluorescence rising complementary area standardized by O-J phase
$N \equiv S_m/ S_S = S_m \bullet M_O \bullet (1/V_J)$	The number of times that Q_A was restored during the period from the start of illumination to FM
$ABS/RC = M_O \bullet (1/V_J) \bullet (1/\varphi_{Po})$	Light energy absorbed by unit reaction center
$\varphi_{Po} \equiv TRO/ABS = [1 - (FO/FM)]$	maximal photochemical efficiency
$\psi_O \equiv ETO/TRO = (1 - VJ)$	The ratio of the excitons captured by the reaction center to the other electron acceptors used to promote electron transfer to the electron transfer chain, which exceeds Q_A, to the excitons used to promote Q_A reduction
$\varphi_{Eo} \equiv ETO/ABS = [1 - (F_O/F_M)] \bullet \psi_O$	Quantum yield for electron transfer
$RC/CSO = \varphi_{Po} \bullet (V_J/M_O) \bullet (ABS/CSO)$	Number of reaction centers per unit area
$PI_{CS} \equiv (RC/CSO) \bullet [\varphi_{Po}/(1 - \varphi_{Po})] \bullet [\psi_O/(1 - \psi_O)]$	Performance index based on unit area

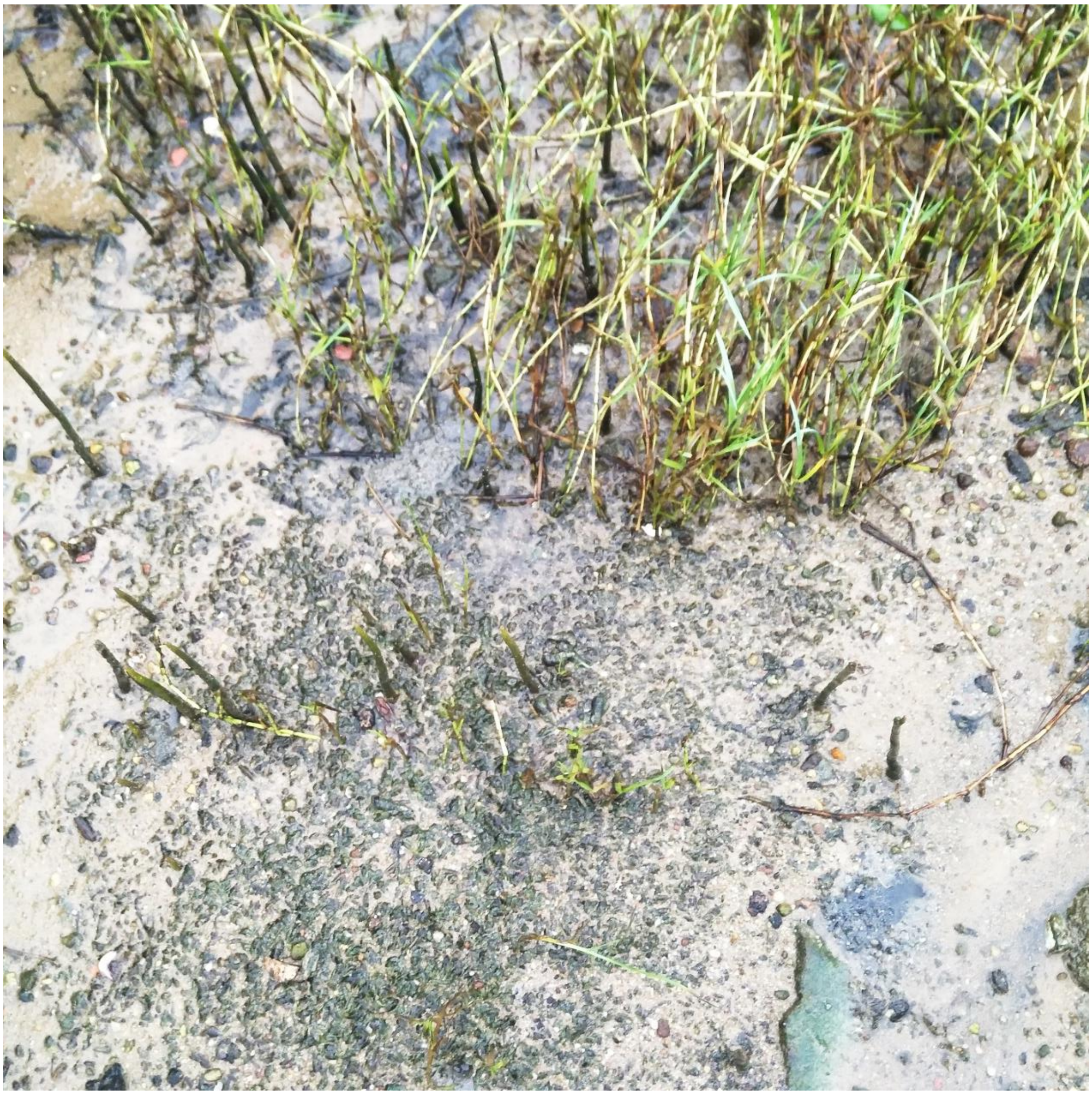

Figure S1. *H. beccarii* associated with the land plant in the high intertidal area

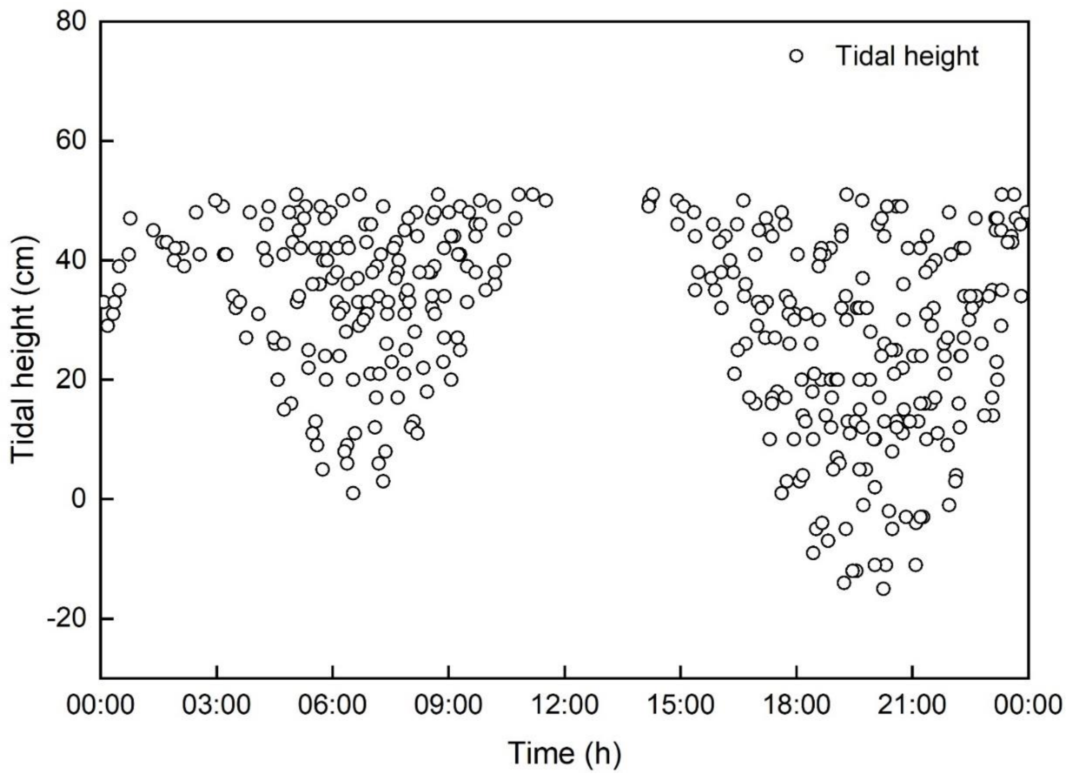

Figure S2. Statistical analysis of the exact timing and tidal height of low tide when seagrasses can emerge in in Yifengxi, along the South China coast, during 2018–2019
